# Adjusting for non-response in the Finnish Drinking Habits Survey

**DOI:** 10.1177/1403494819840895

**Published:** 2019-04-11

**Authors:** Hanna Tolonen, Miika Honkala, Jaakko Reinikainen, Tommi Härkänen, Pia Mäkelä

**Affiliations:** 1 National Institute for Health and Welfare, Department of Public Health Solutions, Finland; 2 Statistics Finland, Finland

**Keywords:** heavy drinking, weighting, non-response, alcohol use

## Abstract

*Aim:* We aim to compare four different weighting methods to adjust for non-response in a survey on drinking habits and to examine whether the problem of under-coverage of survey estimates of alcohol use could be remedied by these methods in comparison to sales statistics. *Method*: The data from a general population survey of Finns aged 15–79 years in 2016 (*n*=2285, response rate 60%) were used. Outcome measures were the annual volume of drinking and prevalence of hazardous drinking. A wide range of sociodemographic and regional variables from registers were available to model the non-response. Response propensities were modelled using logistic regression and random forest models to derive two sets of refined weights in addition to design weights and basic post-stratification weights. *Results*: Estimated annual consumption changed from 2.43 litres of 100% alcohol using design weights to 2.36–2.44 when using the other three weights and the estimated prevalence of hazardous drinkers changed from 11.4% to 11.4–11.8%, correspondingly. The use of weights derived by the random forest method generally provided smaller estimates than use of the logistic regression-based weights. ***Conclusions*: The use of complex non-response weights derived from the logistic regression model or random forest are not likely to provide much added value over more simple weights in surveys on alcohol use. Surveys may not catch heavy drinkers and therefore are prone for under-reporting of alcohol use at the population level. Also, factors other than sociodemographic characteristics are likely to influence participation decisions.**

## Introduction

Response rates are commonly taken in survey research as crude proxies of the level of representativeness of the obtained results to the target population. Declining response rates are a common problem in survey research, putting the representativeness of the survey results in question. When non-response is completely random, that is, not dependent on survey outcome or any of its determinants, even low response rates can provide unbiased, representative results. Unfortunately, survey non-response is usually rather selective: more often, non-respondents tend to be men and represent younger age groups and lower socioeconomic status [1–4]. Because many survey outcomes, such as alcohol use, are known to vary by these factors, this type of selectivity can introduce bias to survey results on alcohol consumption. The selective non-response may be one reason for systematic and gross under-reporting of average consumption levels by surveys in comparison to sales statistics [5].

Previous studies have established a connection between survey non-response and alcohol consumption. In a Dutch survey, it was found that abstainers were over-represented among non-respondents but there was also weak evidence of over-representation of excessive drinking among non-respondents [6]. Similar results have been reported from Norway [7]. Register-linkage studies have shown that in the follow up of survey samples, non-respondents have significantly higher mortality risk from alcohol-related causes than respondents [8, 9]. For example, according to a Finnish study [8], excess mortality of non-respondents from alcohol-related causes was 3.1-fold for men and 4.3-fold for women.

Studies using weighing to adjust for survey non-response have shown moderate impacts. A Canadian study found that after adjusting for survey non-response by age, sex, province and some socioeconomic variables, the prevalence of alcohol use in past 12 months increased by 3.3% (absolute change 2.6 percentage points), chronic risky alcohol use by 13% (absolute change 0.9 percentage points) and heavy monthly alcohol use by 4.3% (absolute change 0.9 percentage points) [10]. A study from New Zealand showed the prevalence of binge drinkers was underestimated by the survey and adjusting for non-response by population weights based on age, sex and area deprivation did not remove underestimation completely [11].

In the Finnish Drinking Habits Surveys carried out in 2000 and 2016, a relatively wide selection of register-based data on the characteristics of respondents and non-respondents was available. The data from 2000 were used to adjust for non-response more thoroughly than just using basic weights typically calculated by a few variables such as sex, age and region. In this survey from 2000, where the response rate was 78%, extended adjustment did not have much further impact on the results [12]. However, this could be a function of the high response rate. Therefore, the current survey with 60% response rate could produce different results. Our aim is to use adjustment weights obtained using different methods and to evaluate how much different adjustment weightings would change the alcohol consumption estimates.

## Methods

### Data

The data came from the Finnish Drinking Habits Survey, a general population survey of Finns aged 15–79 years in the autumn of 2016. The sample was drawn from the national Population Information System (http://vrk.fi/en/population-information-system) using simple random sampling excluding the Åland Islands (0.5% of the population), and the homeless and institutionalized (1.5%). Young adults aged 18–29 were given a two-fold selection probability in the sampling compared to other age groups, which is included in the design weights. The survey was carried out by Statistics Finland as face-to-face interviews. The response rate was 60%.

The study protocol was approved by the ethics committees of the National Institute for Health and Welfare and Statistics Finland. Access to non-respondents’ data was only available to the team member employed by Statistics Finland (MH), who carried out the statistical analyses.

### Measurement

Two outcome measures were an annual volume of drinking and a prevalence of hazardous drinking.

Volume of drinking over the previous 12 months was derived from the so-called “survey period” measure, which is calculated on the basis of the amounts consumed on *all* drinking occasions that occurred in a specified period of time preceding the interview. The covered period ranged from 1 week for most frequent drinkers to 12 months for least frequent drinkers. The length of the period was chosen to cover four drinking occasions as an expected number. The volume consumed in the survey period in centilitres of 100% alcohol was scaled into a year by multiplying with a constant (e.g. multiplied with 52 for a 1-week period). If the period had been restricted to 1 week for all respondents, the level of the estimated volume of drinking would have increased by about one-quarter. The whole survey period measure was used in the analysis because of comparability to previous estimates, lower random variation for occasional drinkers and because the level of the estimate is not expected to influence the comparison of the impacts of different weights.

*Hazardous drinking* was measured using the Alcohol Use Disorders Identification Test (AUDIT) developed by the World Health Organization [13]. The cut-point used to indicate hazardous drinking was 11 on the AUDIT score.

Variables used to adjust for non-response were obtained from administrative registers of Statistics Finland. These included: sex, age group, marital status (unmarried, married or in a registered relationship, other), language (Finnish, Swedish, other), municipality type (rural, semi-urban, urban), educational achievement (basic, secondary and tertiary), socioeconomic status (upper non-manual, lower non-manual, manual employees, other), urban-rural division based on geographical data (core urban area, intermediate zone between rural and urban areas, rural area), individual taxable income (below €20,000, €20,000–40,000, over €40,000; and in deciles divided to 1, 2–5, 6–9, 10), number of under-aged children living in same residence (none, 1–2, 3 or more) as well as their age (none, youngest 0–6 years, other), and region (capital region, other Uusimaa, Southern Finland, Western Finland, Eastern and Northern Finland combined).

### Statistical methods

Both outcome measures were calculated using four different sets of weights: design weights, basic weights, and two more refined weights derived using logistic regression [14] or random forest [15] models. The *design weights* were calculated by dividing the population in three age groups (15–17, 18–29 and 30–79) by their sample sizes.

To derive the *basic weights*, we first calculated post-stratification weights by dividing the population into three age groups by the number of respondents. Then the post-stratification weights were calibrated to match the following population marginal distributions: *region* (six classes) and interaction between *sex and age group* (15–17, 18–24, 25–29, 30–39, 40–54, 55–69, 70–79). The calibration was carried out with Calmar 2 program [16].

The two *refined weight variables* were derived in three phases. First, post-stratification weights were calculated similarly as for the basic weights. Second, response propensities were estimated using two separate models and the post-stratification weights were divided by these two sets of response propensities. Third, the weights were calibrated similarly as for the basic weights. Only the second phase varied between the two refined-weight variables.

When estimating the response propensities, a binary response indicator (1 = respondent, 0 = non-respondent) was the dependent variable. As explanatory variables, we used the register variables that were found to be associated with the response indicator.

The logistic regression model included following categorical variables as explanatory variables: *age group, marital status, language, municipality type, educational achievement, socioeconomic status, urban-rural division based on geographic data* and *individual taxable income (EUR)*. The model also included following interactions: age group * socioeconomic status, age group * marital status, age group * individual taxable income (EUR), age group * language and marital status * municipality type. The variable selection was based on Akaike information criterion. The model produced individual response propensities. The weights took into account the variables and interactions contained in the model.

Random forest is a non-parametric machine-learning method, which tries to find automatically possible interactions and non-linearities. Our random forest consisted of 1000 classification trees based on bootstrap samples. Each tree was based on the same main effect variables as the logistic regression model and additionally the following explanatory variables: *sex, under-aged children living in the same residence (two different variables), region* and *individual taxable income (in deciles).* The variables were selected using the minimal depth method. Random forest was implemented using the R-package randomForestSRC [17].

## Results

Average annual consumption for the whole population was with design weights 2.43 litres of 100% alcohol, with basic weights 2.44, with random forest weights 2.36 and with logistic regression weights 2.42 (Table I). Taking into account that registered per capita consumption (official sales statistics) for 15 years and older population in 2016 was 8.4 litres of 100% alcohol, the impact of any weighting has to be considered small. The weighting did not, on average, contribute to explaining the underestimation of average annual consumption.

**Table I.** Average annual consumption (centilitres of 100% alcohol).

Population group		Responses		Design weight	Weighted								
		(%)	*N*		Basic weights			Random forest			Logistic regression		
					Estimate	Difference^a^		Estimate	Difference^a^		Estimate (95% CI)	Difference^a^	
						Absolute	%		Absolute	%		Absolute	%
All		60%	2285	242.5	244.4	1.9	0.8%	236.3	–6.2	–2.6%	241.9 (224.9, 258.9)	–0.6	–0.2%
Sex	Men	60%	1171	359.7	365.7	6.0	1.6%	353.0	–6.7	–1.9%	360.8 (331.0, 390.7)	1.1	0.3%
	Women	60%	1114	120.9	125.2	4.3	3.4%	121.7	0.8	0.7%	125.1 (111.0, 139.1)	4.2	3.4%
Age group (years)	15–24	54%	457	228.1	231.6	3.5	1.5%	228.7	0.6	0.3%	235.4 (203.7, 267.2)	7.3	3.1%
	25–39	58%	570	290.1	291.9	1.8	0.6%	275.6	–14.5	–5.3%	292.3 (254.1, 330.5)	2.2	0.8%
	40–54	58%	473	274.1	273.2	–0.9	–0.3%	271.1	–3.0	–1.1%	268.7 (231.2, 306.3)	–5.4	–2.0%
	55–79	67%	785	202.5	200.0	–2.5	–1.3%	191.1	–11.4	–6.0%	194.3 (167.6, 221.0)	–8.2	–4.2%
Educational level	Low	54%	588	195.2	201.9	6.7	3.3%	193.5	–1.7	–0.9%	201.4 (170.9, 231.8)	6.2	3.1%
	Middle	58%	1007	267.7	268.2	0.5	0.2%	260.7	–7.0	–2.7%	267.6 (240.7,2 94.5)	–0.1	–0.0%
	High	70%	690	247.0	246.8	–0.2	–0.1%	240.8	–6.2	–2.6%	244.3 (213.2, 275.5)	–2.7	–1.1%

a Difference calculated between design weight and weight in question.

CI: confidence interval.

The use of weights derived by the random forest method provided smaller estimates for all population sub-groups except for women and 15 to 24-year-old people. Two other weights, basic weights and weights derived by logistic regression, provided slightly higher average annual consumption for most population sub-groups, but observed differences compared to results obtained using design weights alone were still small (within 0.0% to 6.0%).

The proportion of hazardous drinking based on AUDIT was 11.4% with design weights, 11.7% with basic weights, 11.4% with random forest weights and 11.8% with logistic regression weights (Table II), so the impact of weighting was relatively small. Similar to the estimate for average annual consumption, weights derived by random forest methods tended to provide lower estimates than design weights. Again, basic weights and weights derived by logistic regression provided marginally higher estimates for most of the population sub-groups. The largest increase in the prevalence estimate was observed among loweducated people with all weights (4.8% with basic and random forest weights and 6.3% with logistic regression weights).

**Table II.** Proportion (%) of hazardous drinking based on audit.

Population group		Design weight	Weighted								
			Basic weights			Random forest			Logistic regression		
			Estimate	Difference^a^		Estimate	Difference^a^		Estimate (95% CI)	Difference^a^	
				Absolute	%		Absolute	%		Absolute	%
All		11.4	11.7	0.3	2.6%	11.4	0.0	0.0%	11.8 (10.4, 13.2)	0.4	3.4%
Sex	Men	16.9	17.5	0.6	3.4%	17.3	0.4	2.3%	17.5 (15.1, 19.9)	0.6	3.4%
	Women	5.9	6.0	0.1	1.7%	5.8	–0.1	–1.7%	6.2 (4.7, 7.6)	0.3	4.8%
Age group (years)	15–24	17.9	18.2	0.3	1.6%	17.7	–0.2	–1.1%	18.7 (15.0, 22.5)	0.8	4.3%
	25–39	15.4	15.5	0.1	0.6%	14.5	–0.9	–6.2%	15.6 (12.2, 19.0)	0.2	1.3%
	40–54	12.3	12.3	–0.0	–0.0%	12.9	0.6	4.7%	12.3 (9.2, 15.4)	0.0	0.0%
	55–79	6.6	6.3	–0.3	–4.8%	6.3	–0.3	–4.8%	6.3 (4.6, 8.1)	–0.3	–4.8%
Educational level	Low	11.8	12.4	0.6	4.8%	12.4	0.6	4.8%	12.6 (9.7, 15.4)	0.8	6.3%
	Middle	13.6	13.8	0.2	1.4%	13.3	–0.3	–2.3%	13.7 (11.4, 16.0)	0.1	0.7%
	High	8.3	8.5	0.1	1.2%	7.9	–0.5	–6.3%	8.4 (6.2, 10.6)	0.0	0.0%

a Difference calculated between design weight and weight in question.

CI: confidence interval.

For both outcomes, estimates obtained with weights derived using different methods (random forest versus logistic regression versus basic post-stratification of much fewer variables) were surprisingly similar. Estimates derived using weights from logistic regression were somewhat higher than those derived using random forest-based weights, but the latter were well within the 95% confidence intervals of the former, that is, the differences were not statistically significant.

## Discussion

As our results have shown, there is a major difference in the alcohol consumption estimates based on surveys and official consumption statistics. The so-called “survey period measure” of the volume of alcohol consumption only covered around 30% of all alcohol consumption in statistics. The significant underestimation was not remedied by any of the used weights; on the contrary, the weights derived by the random forest method increased the difference. This implies that non-response to the Finnish Drinking Habits Survey is not determined by socioeconomic position or any of the variables used in our models. There may be other factors such as attitudes, health behaviours, lifestyles, dislike of surveys or authorities and so on, which may play a more important role in the decision to participate. It also may be that surveys do not catch heavy users, which means we are not able to build a correct profile of heavy users. This would influence the validity of our weights. This is supported by another study from Finland that has used follow-up data of alcohol-related hospitalizations and deaths as proxy indicators for heavy alcohol use in Bayesian modelling [18]. In this study, the prevalence of heavy drinking was 1.5 times higher among men and 1.8 times higher among women, after adjusting for non-response. This shows that non-response is an important reason for the underestimation of the prevalence of heavy drinking.

Unfortunately, data on hospitalizations and mortality that could be individually linked to the entire survey sample, namely, both respondents and non-respondents, are only rarely available for researchers to make this type of adjustment. Even if this would be technically possible, informed consent from the respondents would be needed, which could lower the response rate and introduce further response bias.

In addition to underestimation, which relates to the selective non-response and our ability to capture that effect, one central explanation for difference between survey estimates and official sales statistics is due to underreporting (measurement error) of alcohol consumption by survey respondents. One sign of this is that question formulation affects the magnitude of under-reporting: when respondents are asked about their alcohol consumption “yesterday”, which removes recall bias, the comparison to the official sales data improves [19]. Also, the mode of questionnaire administration (self-administered versus interview) impacts results, suggesting that bias related to denial and concealment are also involved [5].

It is important to use design weights for survey results to adjust for survey design especially when there has been oversampling of some population groups. However, based on our results, further weighting for non-response did not change the results significantly. Similar results were obtained previously from the Finnish Alcohol Habit Survey in 2000 with a higher response rate [12]. Therefore, the use of complex non-response weights derived from the logistic regression model or the random forest are not likely to provide much added value over more simple weights.

## References

[1] Ekholm O Gundgaard J Rasmussen NK et al The effect of health, socio-economic position, and mode of data collection on non-response in health interview surveys. Scand J Public Health 2010;38:699–706. 20851845 10.1177/1403494810382474

[2] Thygesen LC Johansen C Keiding N et al Effects of sample attrition in a longitudinal study of the association between alcohol intake and all-cause mortality. Addiction 2008;103:1149–1159. 18554348 10.1111/j.1360-0443.2008.02241.x

[3] Tolonen H Laatikainen T Helakorpi S et al Marital status, educational level and household income explain part of the excess mortality of survey non-respondents. Eur J Epidemiol 2010;25: 69–76. 19779838 10.1007/s10654-009-9389-9

[4] Reinikainen J Tolonen H Borodulin K et al Participation rates by educational levels have diverged during 25 years in Finnish health examination surveys. Eur J Public Health 2018;28:237–243. 29036286 10.1093/eurpub/ckx151

[5] Gmei G Rehm J. Measuring alcohol consumption. Contemp Drug Prob 2004;31:467–540.

[6] Lahaut VMHCJ Jansen HAM van de Mheen D et al Non-response bias in a sample survey on alcohol consumption. Alcohol Alcoholism 2002;37:256–260. 12003914 10.1093/alcalc/37.3.256

[7] Torvik FA Rognmo K Tambs K. Alcohol use and mental distress as predictors of non-response in a general population health survey: the HUNT study. Soc Psychiatry Psychiatr Epidemiol 2012;47:805–816. 21544604 10.1007/s00127-011-0387-3 PMC3328681

[8] Jousilahti P Salomaa V Kuulasmaa K et al Total and cause specific mortality among participants and non-participants of population-based health surveys: A comprehensive follow up of 54 372 Finnish men and women. J Epidemiol Community Health 2005;59:310–315. 15767385 10.1136/jech.2004.024349 PMC1733044

[9] Gorman E Leyland AH McCartney G et al Adjustment for survey non-representativeness using record-linkage: Refined estimates of alcohol consumption by deprivation in Scotland. Addiction 2017;112:1270–1280. 28276110 10.1111/add.13797 PMC5467727

[10] Zhao J Stockwell T Macdonald S. Non-response bias in alcohol and drug population surveys. Drug Alcohol Rev 2009;28:648–657. 19930019 10.1111/j.1465-3362.2009.00077.x

[11] Meiklejohn J Connor J Kypri K. The effect of low survey response rate on estimates of alcohol consumption in a general population survey. PLoS One 2012;7:e355357. 10.1371/journal.pone.0035527 PMC3332039 22532858

[12] Mäkelä P. Impact of correcting for nonresponse by weighting on estimates of alcohol consumption. J Stud Alcohol 2003;64:589–596. 12921202 10.15288/jsa.2003.64.589

[13] Saunders JB Aasland OG Babor TF et al Development of the alcohol use disorders identification test (AUDIT): WHO Collaborative project on early detection of persons with harmful alcohol consumption - II. Addiction 1993;88: 791–804. 10.1111/j.1360-0443.1993.tb02093.x 8329970

[14] Carson B Williams S. A comparison of two methods to adjust weights for non-response: Propensity modeling and weighting class adjustments. Proceedings of the Annual Meeting of the American Statistical Association 2001 http://www.asasrms.org/Proceedings/y2001/Proceed/00111.pdf

[15] Breiman L. Random forests. Machine Learning 2001;45:5–32.

[16] Sautory O. Carmer 2: A new version of the Calmar calibration adjustment programme. Proceedings of Statistics Canada’s Symposium 2003 https://www.statcan.gc.ca/english/freepub/11-522-XIE/2003001/session13/sautory.pdf

[17] Ishwaran H Kogalur UB Random forests for survival, regression, and classification (RF-SRC), R. package version 2.5.0. 2017.

[18] Korpa J Mäkelä P Tolonen H et al Follow-up data improve the estimation of the prevalence of heavy alcohol consumption. Alcohol Alcoholism 2018; 53:586-596. 29590313 10.1093/alcalc/agy019

[19] Stockwell T Zhao J Chikritzhs T et al What did you drink yesterday? Public health relevance of a recent recall method used in the 2004 Australian National Drug Strategy Household Survey. Addiction 2008;103:919–928. 18482414 10.1111/j.1360-0443.2008.02219.x PMC2782945

